# Text mining datasets of β-hydroxybutyrate (BHB) supplement products’ consumer online reviews

**DOI:** 10.1016/j.dib.2020.105385

**Published:** 2020-03-06

**Authors:** Ji Li, Dan Lowe, Luke Wayment, Qingrong Huang

**Affiliations:** a Nutraceutical Corporation, Utah, USA; b Department of Food Science, Rutgers, the State University of New Jersey, New Jersey, USA

**Keywords:** Text mining, β-hydroxybutyrate (BHB), Sentiment analysis, Complex analysis

## Abstract

The current dataset is obtained by text mining of β-hydroxybutyrate (BHB) supplement products’ consumer online reviews. The text data of 71 BHB products’ consumer reviews were extracted with the aid of the Web Scraper Chrome extension. Then, a lexicon-based sentiment analysis approach was developed to classify the sentiment or polarity of BHB products’ consumer reviews. Both word-level and sentence-level sentiment analyses were conducted to score the analyzed text snippets. In terms of word-level sentiment analysis, word clouds of selected BHB products’ reviews were generated to give direct observation, and the statistics of high-frequent sentiment words were listed for comparison. In terms of sentence-level sentiment analysis, two factors such as flavor and package were taken into consideration to map the products' polarity distributions. Besides, the complex analysis provides us with the basic statistics of the analyzed BHB customer reviews data.

**Table:** **Specifications Table**

Subject	Medicine and Dentistry
Specific subject area	Health Informatics
Type of data	Table, Figure
How data were acquired	A lexicon-based sentiment analysis approach was developed to classify the sentiment or polarity of β-hydroxybutyrate (BHB) products’ consumer reviews obtained from Amazon.com.
Data format	Raw, Analyzed
Parameters for data collection	The text data of β-hydroxybutyrate (BHB) products’ consumer reviews were extracted with the aid of the Web Scraper Chrome extension. Positive, negative, neutral, and compound scores were assigned to the analyzed text snippets through sentiment analysis. The basic text statistics including number of characters, number of words, number of sentences, and number of unique words in those reviews were obtained through text complexity analysis.
Description of data collection	The entire text data of β-hydroxybutyrate (BHB) products’ consumer reviews on Amazon.com were collected within 2 months of the year 2019. Then, the word-level and sentence-level sentiment analyses were conducted based on the collected text data. Through analysis, the scores such as positive, negative, neutral, and compound were assigned to the analyzed text data. Among them, the compound score gives the overall rating within the range from −100% to +100%. Factors such as flavor and packaging were considered to map the BHB products' polarity distributions. Besides, the complexity analysis was used to provide the text statistics of analyzed BHB product reviews including the word number, sentence number, and character number in the reviews.
Data source location	Amazon.com (an online data source)
Data accessibility	Data is available in the supplementary file attached with this article.

## Value of the Data

• The datasets of text-mining β-hydroxybutyrate (BHB) supplements’ consumer online reviews is a new marketing research of dietary supplements. It helps the researchers, product developers, and marketers in the field of nutrition to develop new healthcare products with affinity to customers.
• The researchers, product developers, and relevant marketing professionals in multiple fields such as functional food, dietary supplement, and nutrition can indirectly or directly benefit from those data.
• Those processed consumers’ feedback data covers the impacts of flavor and packaging upon the consumer acceptance of novel dietary supplements.
• The sentiment analysis used here provides us with an innovative approach to resolve the customer feedbacks upon fast-moving consumer goods (FMCG) products.

## 1 Data

**Fig. 1:**
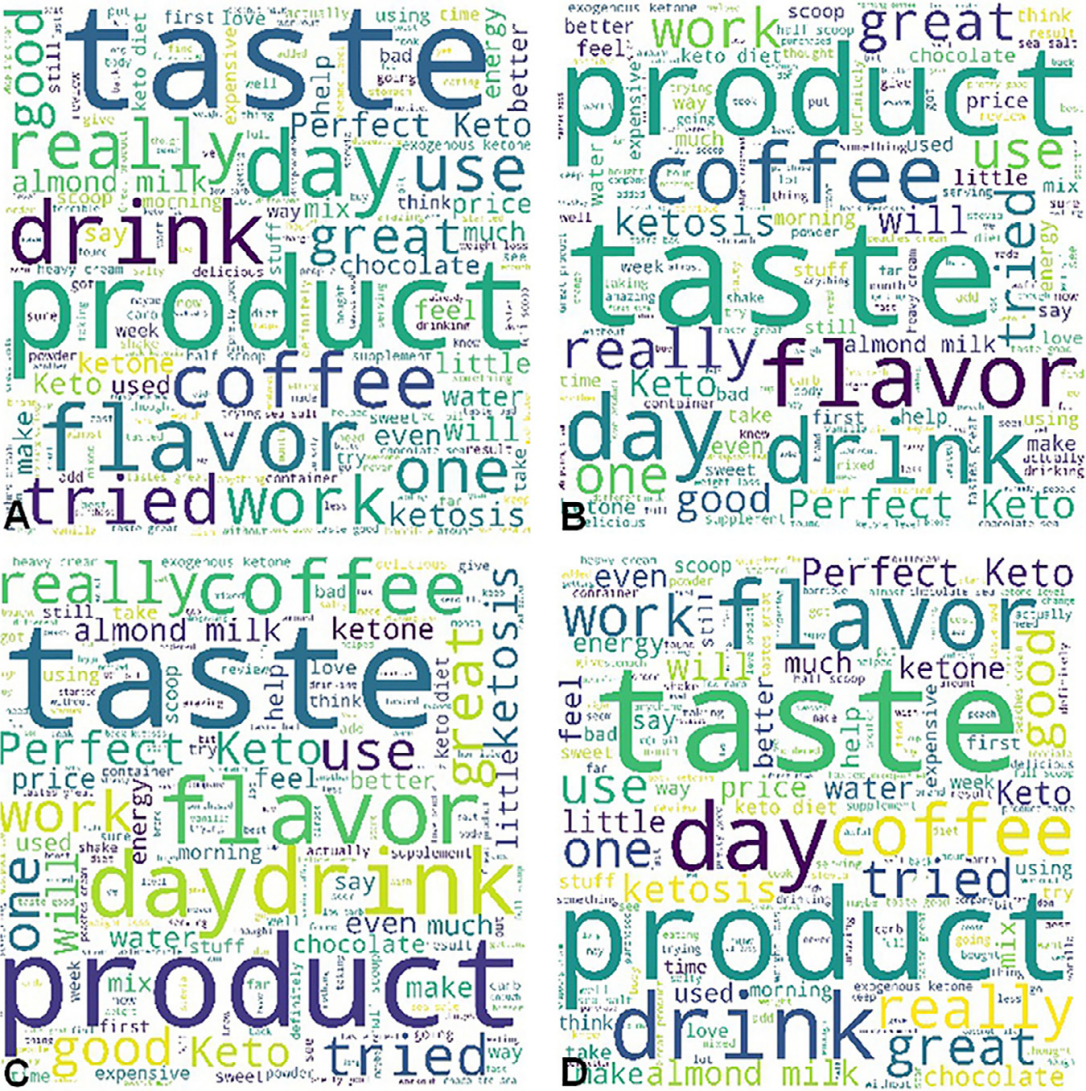
Word clouds of brand A's β-hydroxybutyrate (BHB) powder products flavored with A. Caramel; B. Chocolate; C. Coffee; and D. Vanilla.

**Fig. 2:**
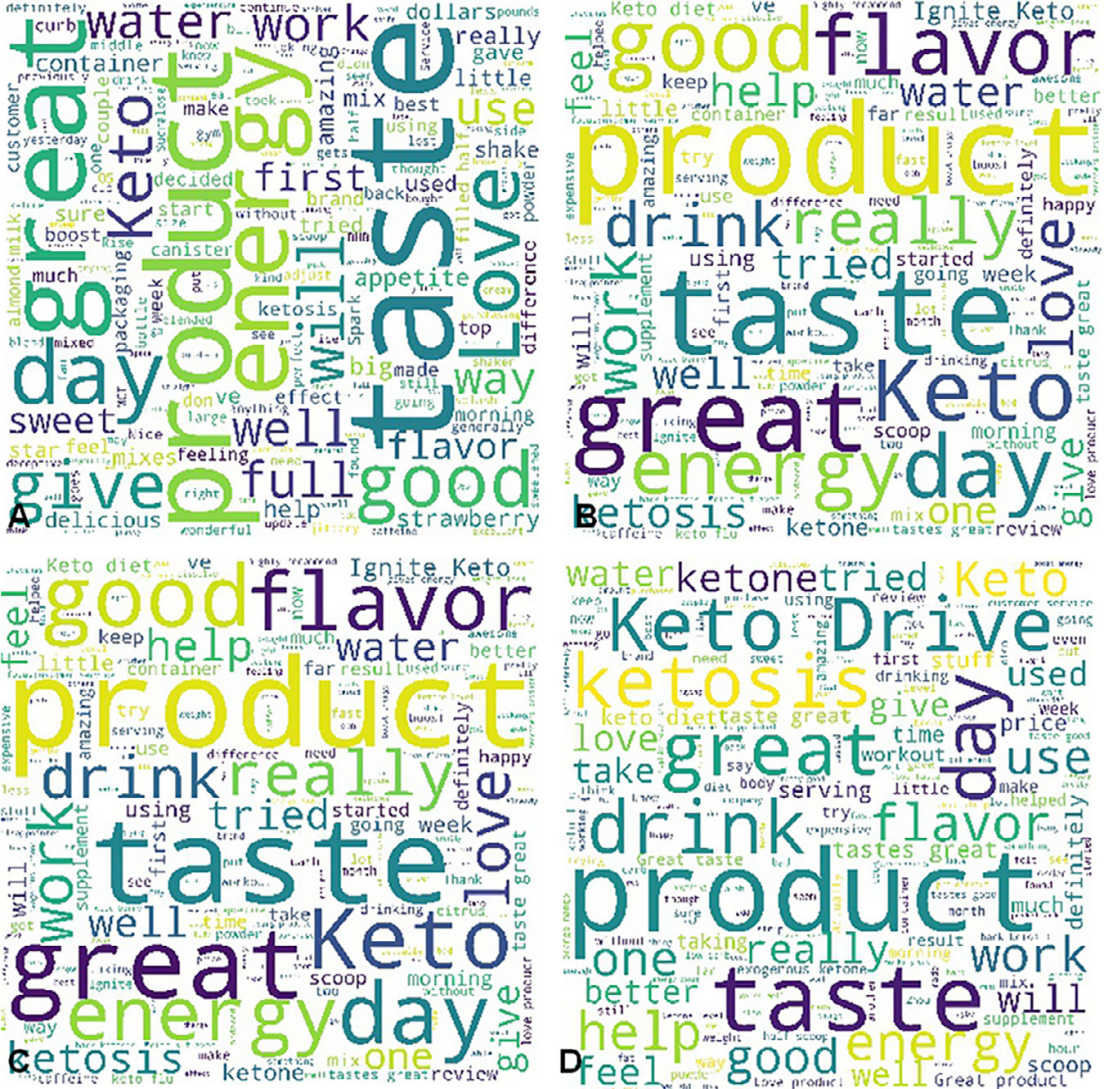
Word clouds of berry flavor involved β-hydroxybutyrate (BHB) powder products under different brands.

**Fig. 3:**
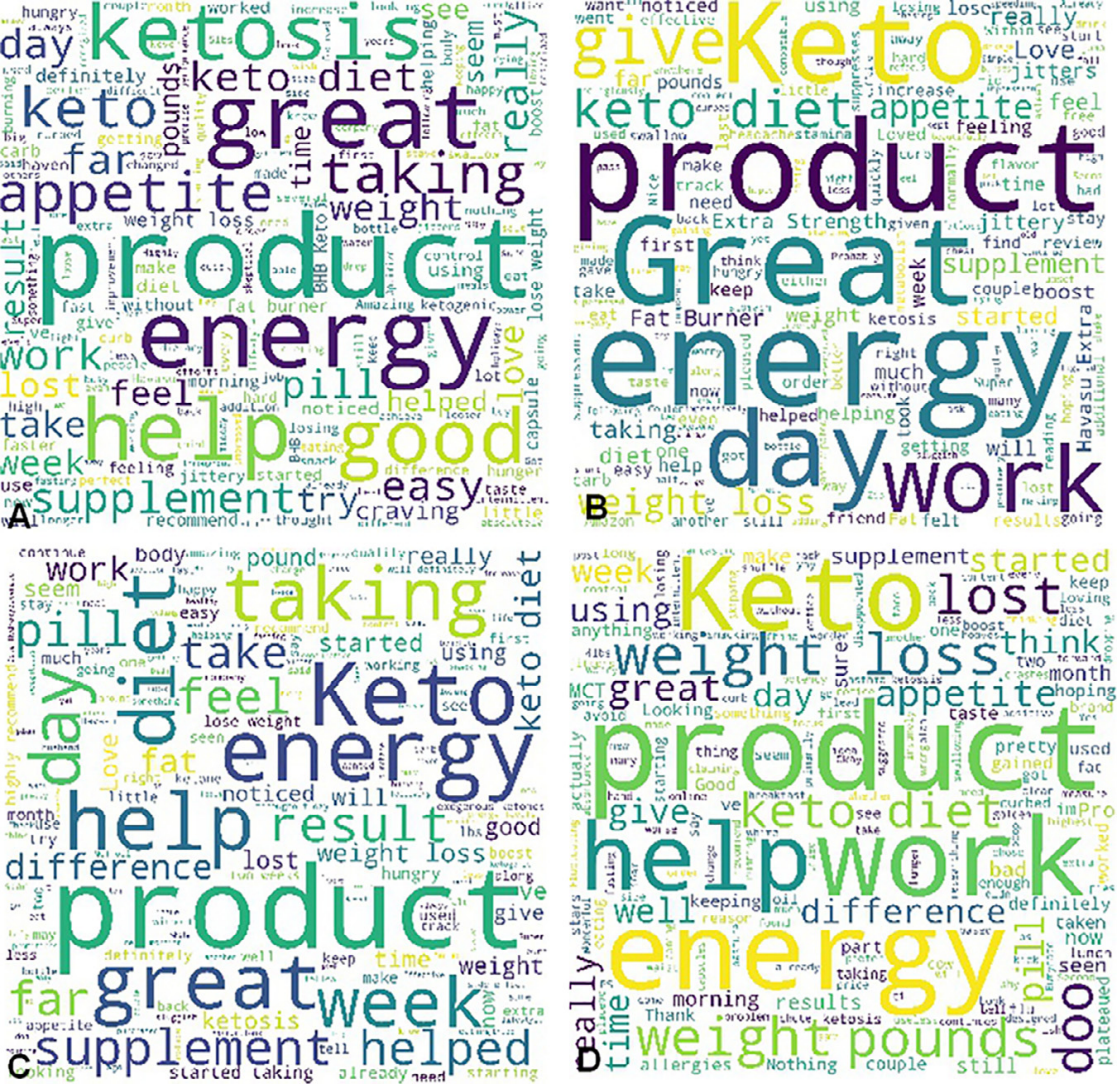
Word clouds of β-hydroxybutyrate (BHB) capsule products under different brands.

**Fig. 4:**
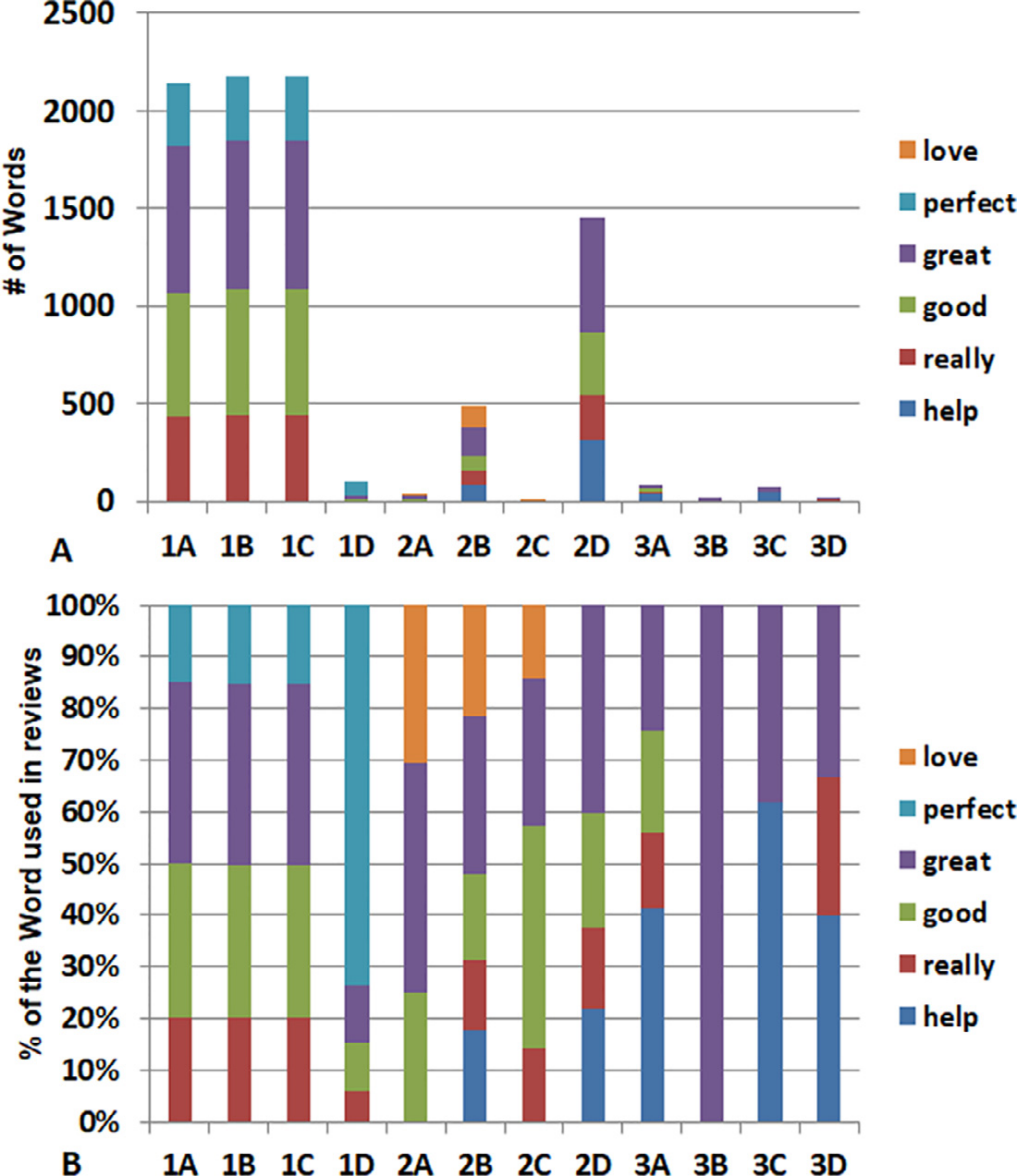
Stack plots of high-frequency sentiment words’ statistics for β-hydroxybutyrate (BHB) powder/capsule products under different brands; BHB 1A indicates BHB product in Fig. 1A.

**Fig. 5:**
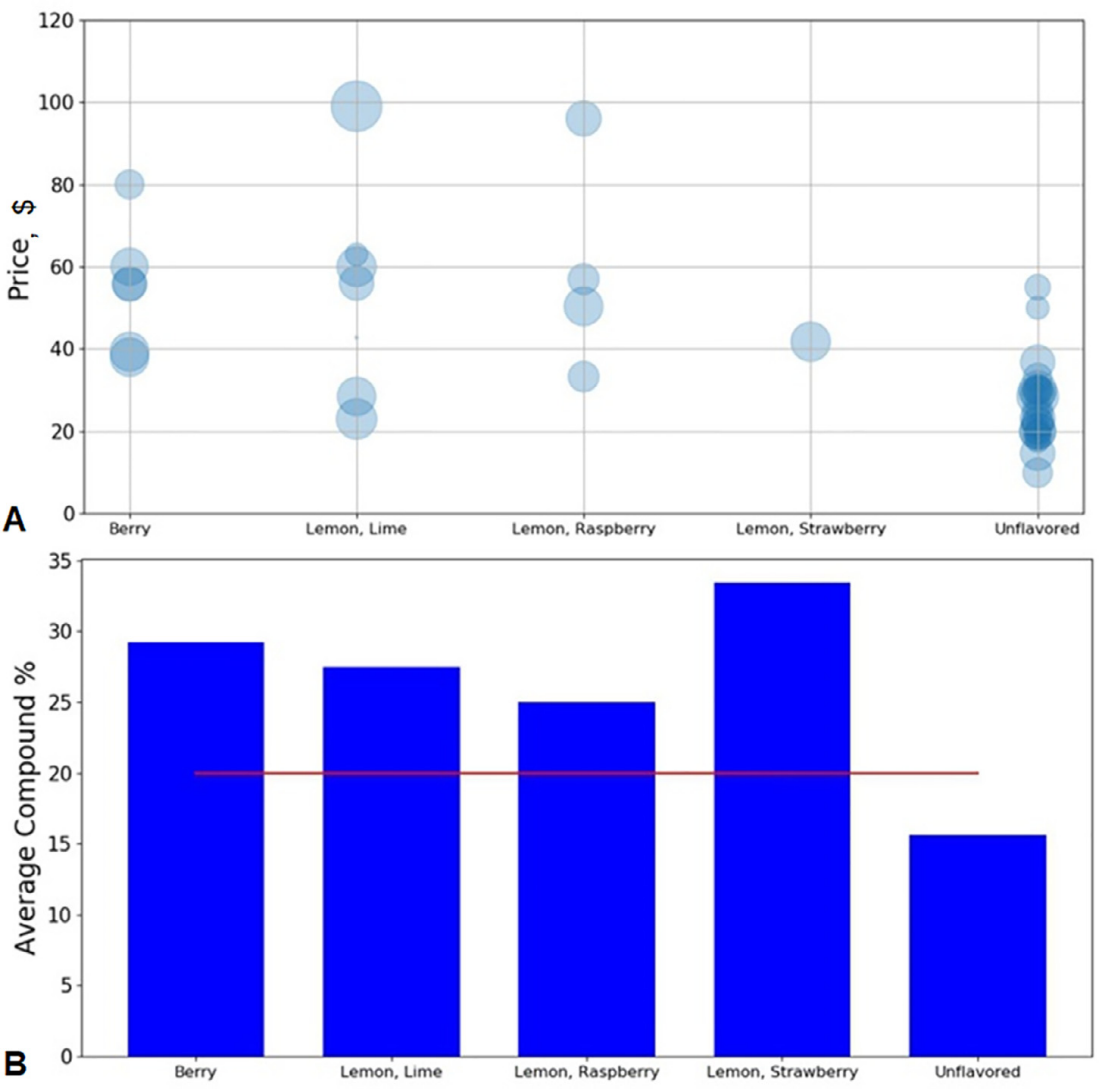
Sentence-level sentiment analysis of β-hydroxybutyrate (BHB) products’ online reviews with berry and lemon-series flavors’ focus. **A** Bubble chart of BHB product price versus flavor; Bubble size indicates the compound score ranging from −100% to +100%; **B** Average compound scores of partially-selected BHB products under berry and lemons-series flavor categories; The line Average Compound = 20% is differentiating line; The item name “Lemon, Lime” indicates the products flavored with lemon, lime, or lemon-lime.

**Fig. 6:**
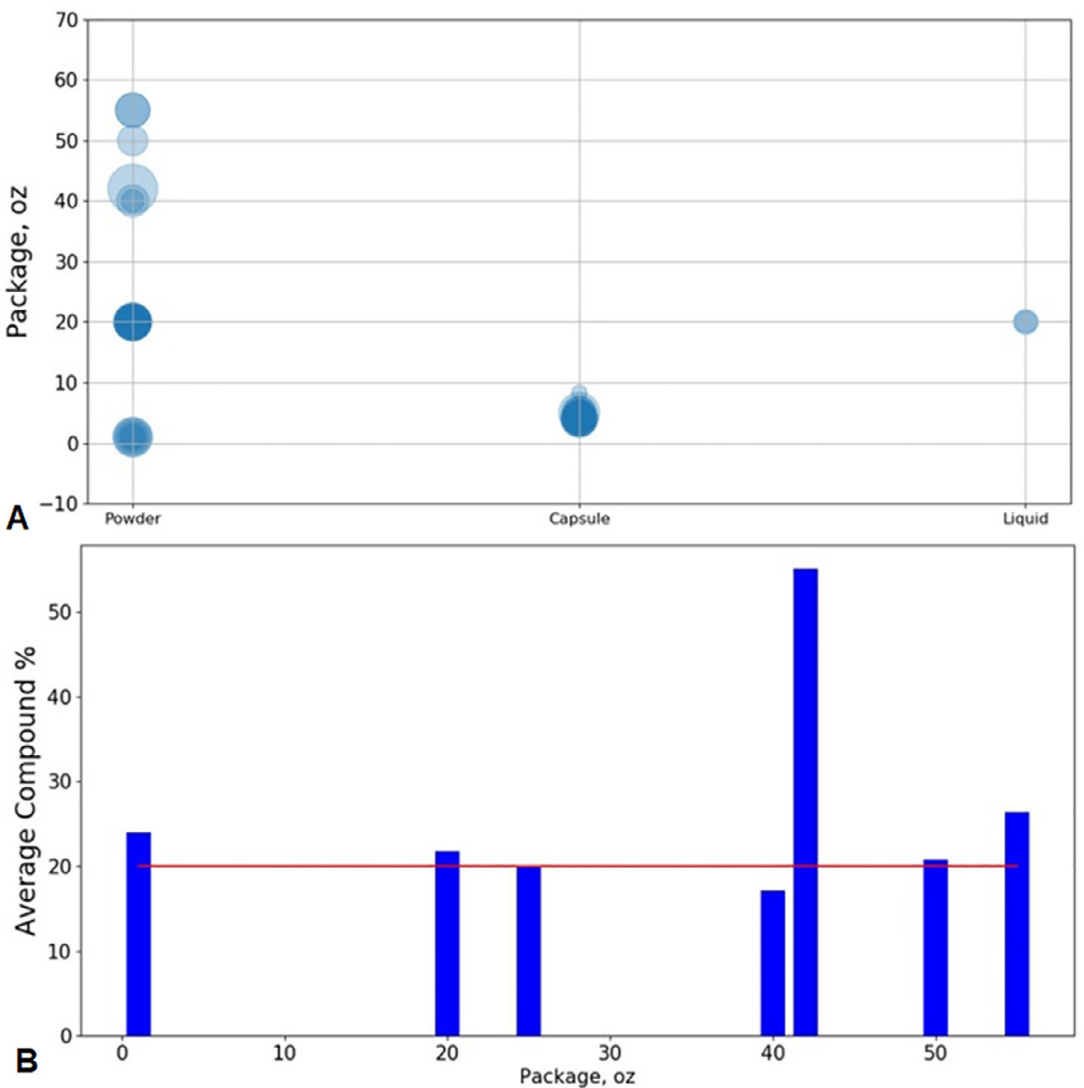
Sentence-level sentiment analysis of BHB products’ online reviews with package focus. **A** Bubble chart of BHB product package versus dosage form; Bubble size indicates the compound score ranging from −100% to +100%; **B** Bar chart of BHB product average compound score versus package; The line Average Compound = 20% is differentiating line.

β-hydroxybutyrate (BHB) is the conjugate base of the organic compound hydroxybutyric acid. Previous studies demonstrated that BHB possessed the functions of stress reduction [1], neural protection [2], seizure alleviation [3], weight loss [4], and body metabolism in starvation [5]. In this investigation, we conducted a lexicon-based sentiment analysis of BHB supplement products’ customer reviews obtained via Amazon.com. The statistics of the entire data pool is summarized in Table 1. For the word-level sentiment analysis, the word clouds of brand A's BHB powder products with different flavors (Fig. 1), berry flavor-involved BHB powder products under different brands (Fig. 2), and BHB capsule products under different brands (Fig. 3) were displayed for direct observation. In addition, the high-frequency sentiment words’ compositions for branded BHB powder/capsule products were shown in Fig. 4. In terms of sentence-level sentiment analysis, two factors such as flavor and packaging were taken into account to map the BHB products’ polarity distributions (Fig. 5A, Fig. 6A), respectively. During sentence-level sentiment analysis, the score assignments such as positive, negative, neutral, and compound for partially-selected BHB products’ consumer reviews were displayed in Table 2. At the same time, the average compound scores in the categories of flavors and packages were calculated and shown in Fig. 5B and Fig. 6B, respectively. Lastly, the complex analysis of partially-selected BHB products were displayed in Table 3.

**Table 1:** Statistics of β-hydroxybutyrate (BHB) products' online review data.

Data Resource	Amazon.com
Typical data	Product review
Data Type	Text
Product Type	Powder, Liquid, Capsule
# of Brands	26
# of Products	71
# of Reviews	30,877
# of Sentences	105,703
# of Words	1,574,171

**Table 2:** Sentence-level sentiment analysis of partially-selected β-hydroxybutyrate (BHB) products’ online reviews.

Product	Dosage	Positive, %	Negative, %	Neutral, %	Compound, %
BHB 1A*	Powder	17.76	7.31	74.84	17.03
BHB 1B	Powder	17.76	7.32	74.83	17.04
BHB 1C	Powder	17.78	7.31	74.83	17.06
BHB 1D	Powder	17.68	7.34	74.89	16.96
BHB 2A	Powder	25.81	3.65	70.54	31.70
BHB 2B	Powder	25.28	5.01	69.71	29.83
BHB 2C	Powder	17.44	10.06	72.50	13.16
BHB 2D	Powder	22.84	5.42	71.70	25.85
BHB 3A	Capsule	24.14	6.22	69.64	27.54
BHB 3B	Capsule	28.36	7.25	64.40	30.78
BHB 3C	Capsule	22.08	4.69	73.23	26.37
BHB 3D	Capsule	20.39	6.90	72.72	17.03

⁎ BHB 1A indicates BHB product in Fig. 1A

**Table 3:** Complex analysis display of partially-selected β-hydroxybutyrate (BHB) products' online reviews.

Product	Dosage	# of Reviews	# of Sentences	# of Words	# of Characters	Len(Vocabulary)
BHB 1A*	Powder	3490	12,940	193,635	889,649	6992
BHB 1B	Powder	3490	12,935	193,636	889,649	6992
BHB 1C	Powder	3490	12,948	193,637	889,649	6992
BHB 1D	Powder	3490	12,942	193,635	889,649	6992
BHB 2A	Powder	52	139	1869	8523	543
BHB 2B	Powder	458	1342	19,177	88,456	1965
BHB 2C	Powder	24	34	487	2293	219
BHB 2D	Powder	1746	5512	81,168	376,469	4553
BHB 3A	Capsule	114	223	3037	14,325	707
BHB 3B	Capsule	58	122	1628	7605	446
BHB 3C	Capsule	145	349	5298	24,714	896
BHB 3D	Capsule	30	89	1153	5212	394

⁎ BHB 1A indicates BHB product in Fig. 1A

## 2 Experimental design, materials, and methods

### 2.1 Online review scrape

The text data of β-hydroxybutyrate (BHB) products’ customer reviews were collected from Amazon.com with the aid of the Web Scraper, a Chrome extension. After collection, text data sometimes require pre-process or cleaning before text mining to minimize the noises or bias [6]. For the reviews in this research, most users express their comments in a brief and straightforward way. There were not many noise and uninformative parts as HTML tags, scripts and advertisements as other online texts [6]. We simply cleaned the text data by removing special characters and reorganizing the content for further analysis. On another side, we tried maintaining the originality of the review contents as much as possible.

### 2.2 Word-level sentiment analysis

An external lexicon served as resource to judge the text sentiment or polarity [7]. The words in online reviews of one product are obtained with NLTK tokenization before sentiment classification [8]. Then, they are classified into categories of positive and negative for further analysis. Besides, word clouds are generated based on the word-tokenized text contents with the wordcloud function in NLTK [8].

### 2.3 Sentence-level sentiment analysis

Vader sentiment analysis of sentence-tokenized text of BHB products’ reviews is performed to gain sentiments such as positive, negative, and polarity scores [9]. This approach provides how positive or negative a snippet under analysis is. In details, the sentence-level snippets are then classified into the categories of positive, negative, neutral, and compound, during which scores are assigned to each snippet. Among the four categories, the compound score measures the sum of all the lexicon ratings (positive, negative, and neutral) that have been normalized between −100% (most extreme negative) and +100% (most extreme positive). The higher the compound score, the more overall positive we obtain.

### 2.4 Text complexity analysis

Text complexity analysis gives a statistical summary of the text data we collected. The text complexity analysis summarizes the number of online reviews for one product, number of characters, number of words, number of sentences, and number of unique words in those reviews. With text complexity analysis, we can take one more dimension to view those text data, judge the text feature, and predict the product market confidently.

### 2.5 Review data summary

Table 1 shows the statistics of BHB product review data collected on Amazon.com. The BHB product reviews in text were collected within 2 months of the year 2019. The entire text data set include 30,877 reviews, 105,703 sentences, and 1,574,171 words. Those product reviews reflect the clients’ feedbacks and comments to 71 products under 26 brands.

## Conflict of Interest

The authors declare that they have no known competing financial interests or personal relationships that could have appeared to influence the work reported in this paper.

## Appendix Supplementary materials

Image, application 1
